# Proteomic Analysis of Fractionated *Eimeria tenella* Sporulated Oocysts Reveals Involvement in Oocyst Wall Formation

**DOI:** 10.3390/ijms242317051

**Published:** 2023-12-02

**Authors:** Liushu Jia, Qiping Zhao, Shunhai Zhu, Hongyu Han, Huanzhi Zhao, Yu Yu, Jia Yang, Hui Dong

**Affiliations:** Key Laboratory of Animal Parasitology of Ministry of Agriculture, Shanghai Veterinary Research Institute, Chinese Academy of Agricultural Sciences, Minhang, Shanghai 200241, China; jialiushu@126.com (L.J.); zqp@shvri.ac.cn (Q.Z.); zhushunhai@shvri.ac.cn (S.Z.); hhysh@shvri.ac.cn (H.H.); zhaohuanzhi225@163.com (H.Z.); yuyuyu20231030@163.com (Y.Y.); dashiliuzi@163.com (J.Y.)

**Keywords:** *Eimeria tenella*, oocyst wall, sporocyst, transmission, proteomics

## Abstract

*Eimeria tenella* is the most pathogenic intracellular protozoan parasite of the *Eimeria* species. *Eimeria* oocyst wall biogenesis appears to play a central role in oocyst transmission. Proteome profiling offers insights into the mechanisms governing the molecular basis of oocyst wall formation and identifies targets for blocking parasite transmission. Tandem mass tags (TMT)-labeled quantitative proteomics was used to analyze the oocyst wall and sporocysts of *E. tenella*. A combined total of 2865 *E. tenella* proteins were identified in the oocyst wall and sporocyst fractions; among these, 401 DEPs were identified, of which 211 were upregulated and 190 were downregulated. The 211 up-regulated DEPs were involved in various biological processes, including DNA replication, fatty acid metabolism and biosynthesis, glutathione metabolism, and propanoate metabolism. Among these proteins, several are of interest for their likely role in oocyst wall formation, including two tyrosine-rich gametocyte proteins (*Et*GAM56, *Et*SWP1) and two cysteine-rich proteins (*Et*OWP2, *Et*OWP6). Concurrently, 96 uncharacterized proteins may also participate in oocyst wall formation. The present study significantly expands our knowledge of the proteome of the oocyst wall of *E. tenella*, thereby providing a theoretical basis for further understanding of the biosynthesis and resilience of the *E. tenella* oocyst wall.

## 1. Introduction

Avian coccidiosis is an intestinal disease caused by infection with several *Eimeria* spp. (protozoa) [1]. Clinical signs included malabsorption, reduced body weight gain, diarrhea, bloody stools, suppression of the immune system, and increased susceptibility to infection with other pathogens [2,3,4]. Coccidiosis is estimated to cost the global poultry industry in excess of US $3 billion per year [5,6]. *Eimeria* spp. undergo asexual reproduction following the invasion of intestinal or other tissue cells by haploid sporozoites. After several cycles of asexual replication, the final generation of merozoites commits to sexual development by forming gametocytes in host cells [7,8]. Unsporulated oocysts are the endpoint of sexual reproduction and are shed via the feces into the environment, become infective (sporulated oocysts), and contaminate food/water supplies, eventually achieving transmission from host to host, which is a critical step for parasite transmission [9,10,11].

A defining characteristic of *Eimeria* spp. is the oocyst, which is notoriously resilient, resisting both mechanical and chemical damage and tolerating changes in humidity and temperature for months [12,13]. What gives rise to oocyst resistance is the oocyst wall, which encapsulates and protects coccidian parasites as they exit their definitive host in feces and, subsequently, in the harsh, external world [14,15]. Studies of the composition and structure of the oocyst wall and the molecular mechanism of oocyst wall biogenesis are the key to understanding the long-term preservation of oocysts in the external environment.

Considerable progress has been made over the past few years towards understanding the molecular basis of oocyst wall formation in *Eimeria* parasites [12,16,17]. In terms of structural composition, previous studies have shown that the oocyst wall is bilayered, consisting of an electron-dense outer layer and an electron-transparent inner layer. At the molecular level, the oocyst wall is made up of more than 90% proteins. Two main types of proteins are involved in the formation of the oocyst wall: tyrosine-rich gametocyte proteins (GAMs), such as GAM56, GAM82, and GAM230 [17,18], and cysteine-rich oocyst wall proteins (OWPs) [19]. However, much work is needed to extend our knowledge of the repertoire of structural proteins and enzymes involved in wall formation.

The aim of this study was to conduct a quantitative proteomics analysis of *Eimeria tenella* oocyst walls and sporocysts. Many novel potential oocyst wall proteins were identified and the implications of our results for our understanding of the molecular basis of oocyst wall formation are discussed.

## 2. Results

### 2.1. Identification of Proteins in Sporulated Oocyst Fractions

**Figure 1:**
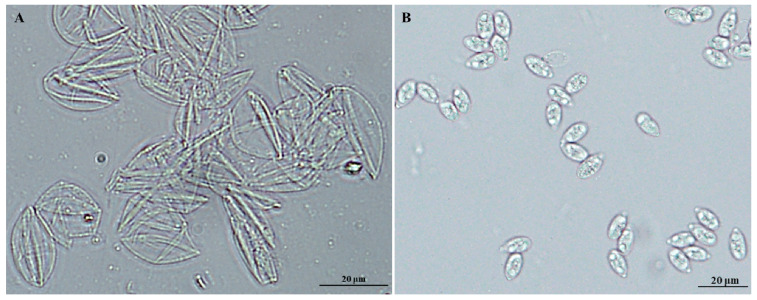
Purified fractions of fragmented oocysts of Eimeria tenella after Percoll gradient centrifugation. Purified sporulated oocyst walls (**A**), and sporocysts (**B**) visualized under Bright-field.

The sporulated oocysts (SO) contain the oocyst walls (SOW) and sporocysts (Spo). We used the Percoll density gradient centrifugation method to isolate the oocyst walls and sporocysts from the purified sporulated oocysts. As shown in Figure 1, there was no oocyst, sporocyst, or other foreign matter in the purified oocyst walls, and there was no oocyst, oocyst wall, or other foreign matter in the purified sporocysts, indicating successful isolation.

**Figure 2:**
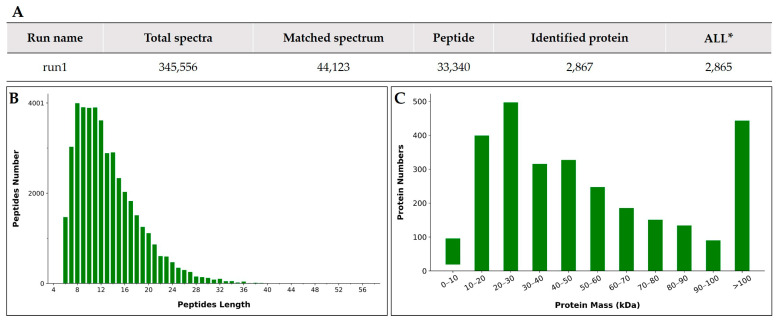
Statistics of root protein identification results: (**A**) Statistics of protein identification results (**B**) Peptides length distribution; (**C**) Proteins molecular weight distribution. All*: Total protein number that can be quantified for all samples.

Quantitative proteomic analyses of proteins from the oocyst wall and sporocyst fractions were performed using the TMT labeling method. We obtained a total of 345,556 spectra and 33,340 peptides, which were mapped to 2867 proteins, among which 2865 had a false discovery rate of <1% (Figure 2A). The details of the identified proteins are shown in Supplementary Table S1. Statistical analysis showed that most of the peptides were between 6 and 24 amino acids long (Figure 2B). In terms of protein mass distribution, a good coverage was obtained for a wide molecular weight range for proteins less than 160 kDa (Figure 2C). The mass tolerance for precursor ions was 10 ppm and the mass tolerance for product ions was 0.02 Da. The accuracy of the mass spectrometer was normal, and the qualitative analysis of the proteins should not be affected by large mass deviations. These results suggest that the obtained data were of relatively high quality and reliable.

**Figure 3:**
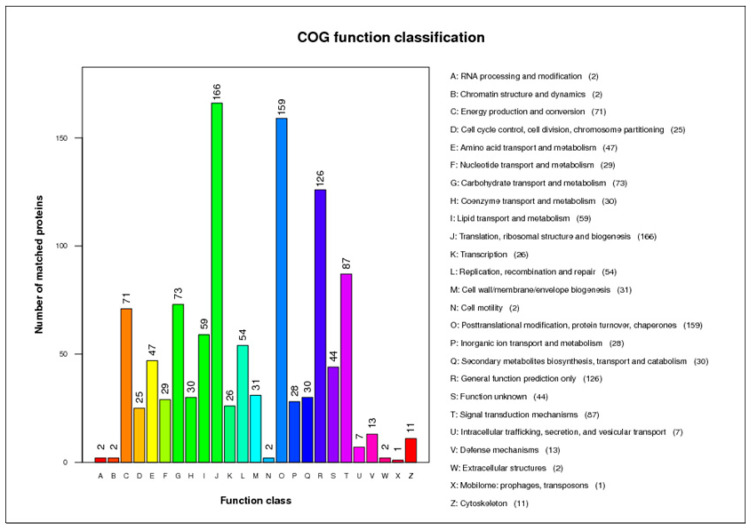
Cluster of Orthologous Groups (COG) analysis of the identified proteins in Eimeria tenella oocyst walls and sporocysts. The y-axis represents the number of proteins. The x-axis represents different COG classes.

The 2865 proteins were analyzed with the COG, and approximately 36.7% were covered and classified into 25 categories (Figure 3). The three categories with the most identified proteins were translation, ribosomal structure, and biogenesis; posttranslational modification, protein turnover, and chaperones and signal transduction mechanisms.

### 2.2. Identification and Functional Analysis of DEPs

**Figure 4:**
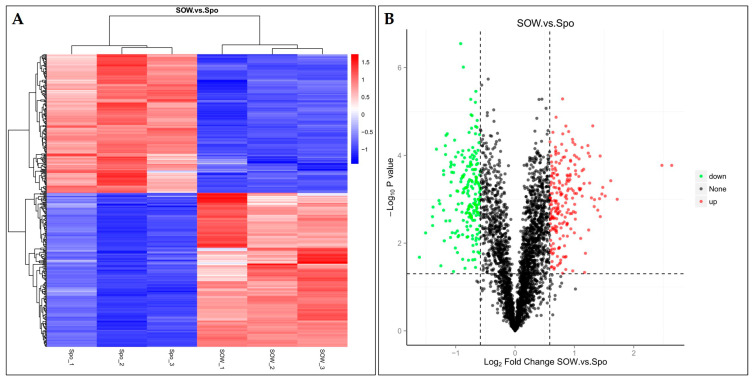
Hierarchical cluster analysis and volcano plot showing DEPs between SOW and Spo fractions. (**A**,**B**) SOW vs. Spo group.

The hierarchical cluster analysis and volcano plots of DEPs between the SOW and Spo fractions are shown in Figure 3. A total of 401 DEPs, of which 211 were upregulated (FC ≥ 1.5, *p* ≤ 0.05) and 190 were downregulated (FC ≤ 0.67, *p* ≤ 0.05), were identified (Figure 4A,B) (Supplementary Table S2).

**Figure 5:**
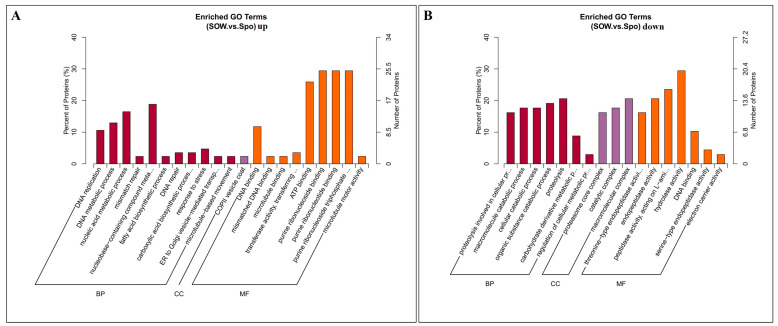
Gene ontology (GO) classification of DEPs. The number of genes with GO−terms in the categories “Biological Process”, “Cellular component”, and “Molecular function” were shown. (**A**,**B**) show the GO−terms of DEPs that are up-regulated and down-regulated in the oocyst wall fractions, respectively.

To further study the functions of the proteins from the oocyst wall and sporocyst fractions, a Gene Ontology enrichment analysis was conducted. The 211 upregulated DEPs were annotated in the biological process, cellular component, and molecular function categories (Figure 5A). The most prevalent biological processes were the nucleobase-containing compound metabolic process (16 proteins), nucleic acid metabolic process (14), and DNA metabolic process (11). The most prevalent cellular component was the COPII vesicle coat (2). The most predominant molecular functions were purine ribonucleoside binding (25), purine ribonucleoside triphosphate binding (25), purine ribonucleotide binding (25), ATP binding (22), and DNA binding (10). Similarly, among the 190 downregulated DEPs, in the “biological process” category the terms proteolysis (14), organic substance catabolic process (13), cellular catabolic process (12), and macromolecule catabolic process (12) were enriched, in the “cellular components” category the terms macromolecular complex (14) and catalytic complex (12) were enriched, and in the “molecular function” category the terms hydrolase activity (20), peptidase activity-acting on L-amino acid peptides (16), and endopeptidase activity (11) were enriched (Figure 5B).

**Figure 6:**
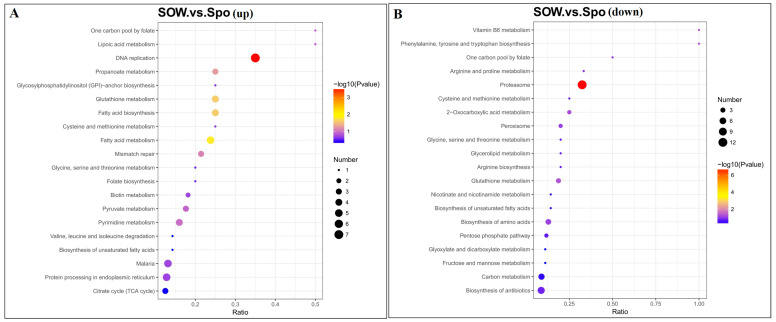
Scatterplot of the top 20 enriched KEGG pathways. Rich factor represents the ratio of the number of DEPs and the number of all protein in the pathways. KEGG pathway enrichment of up−regulated (**A**) and down−regulated (**B**) DEGs in SOW vs. Spo group.

KEGG pathway enrichment analysis of DEPs was conducted (Figure 6). The upregulated DEPs were involved in DNA replication, fatty acid metabolism, fatty acid biosynthesis, glutathione metabolism, and propanoate metabolism (Figure 6A). In addition, the down-regulated DEPs were associated with the proteasome, glutathione metabolism, carbon metabolism, biosynthesis of antibiotics, the pentose phosphate pathway, etc. (Figure 6B).

### 2.3. Most Abundant Up- and Down-Regulated Proteins in Oocyst Walls

The 211 upregulated DEPs comprised 115 previously described proteins and 96 uncharacterized proteins. The top 30 proteins with the highest abundance in oocyst walls are listed in Table 1. Equisetin synthetase, amiloride-sensitive amine oxidase, GPI transamidase subunit PIG−U, penicillin amidase domain−containing protein, and oocyst wall protein were the most abundant proteins. In addition, 14 proteins involved in the biosynthesis of oocyst walls (Table 2), 19 enzymes (Table 3), and 10 common functional proteins (Table 4) were found in upregulated DEPs.

The 190 downregulated DEPs comprised 94 previously described proteins and 96 hypothetical proteins. Among the upregulated DEPs in the sporocyst fraction, 14 interesting proteins were identified, such as a SAG family member, heat shock protein, micronemal proteins, and several protein kinases (Table 5).

### 2.4. Validation of TMT Data for Selected Proteins by Western Blot Assay

**Figure 7:**
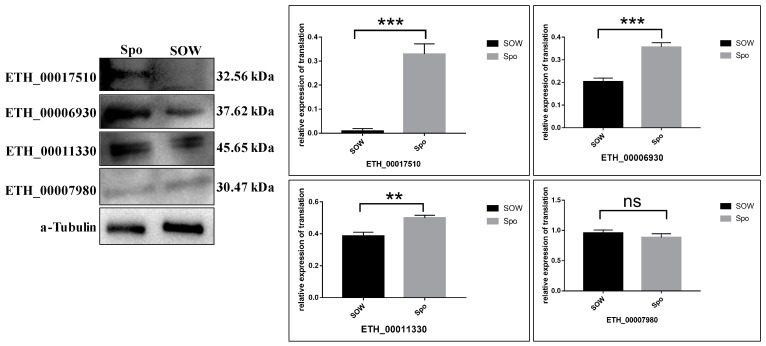
Verification of the protein expression by Western blot. Four proteins were randomly selected from laboratory protein antibodies for validation of Proteomics data. ETH_00017510, ETH_00006930, ETH_00011330, and ETH_00007980 represent the proteins heat shock protein, Microneme protein Etmic-2, SERPIN1 protein, and 14-3-3 protein. α-tubulin was used for reference protein. ** indicated a significant difference (*p* < 0.01), *** shown extremely significant difference (*p* < 0.001), ns was nonsignificant (*p* > 0.05).

The protein expression levels obtained by the TMT labeling assay were further confirmed by quantifying the expression levels of five proteins by western blot. As shown in Table 6 and Figure 7, the trends of four DEPs were generally consistent with the TMT results, while we failed to detect acetyl-CoA carboxylase (ETH_00009260). This may be due to the fact that cross-linking of the cysteine-rich protein during oocyst wall assembly drastically reduced the affinity of this particular antibody, although the reduction and alkylation performed prior to western blot analysis should have been sufficient for breaking disulfide bonds [20].

## 3. Discussion

Among the *Eimeria* species causing chicken coccidiosis, *E. tenella* is the most pathogenic intracellular protozoan parasite. The high prevalence of *E. tenella* can be attributed to the resilient oocyst wall, which protects the sporozoites from desiccation and chemical disinfection in the external environment, facilitating transmission from host to host [21]. Due largely to difficulties in isolating large amounts of oocyst walls, relatively little is known about how this structure is able to resist extreme environmental stresses. In the present study, highly purified oocyst walls and sporocysts were isolated successfully from disrupted sporulated oocysts of *E. tenella* with a revised Percoll density gradient centrifugation method described by Han et al. (2010) and used to comprehensively elucidate the protein expression profiles by TMT quantitative proteomics. The results of our proteomic analysis showed that a total of 401 proteins were significantly differentially expressed between the oocyst wall and sporocysts. As we want to understand the molecular basis of oocyst wall formation, the following discussion is mainly focused on the 211 up-regulated DEPs in oocyst walls.

The oocyst wall of *Eimeria* is primarily made up of proteins (>90%), but so far, only two types of oocyst wall proteins have been identified [22]. The best characterized of these are the tyrosine-rich gametocyte proteins GAM56, GAM82, and GAM230, localized to the wall forming body II (WFBII) of mature macrogametes and to the inner layer of the oocyst wall [16,17]. In the present study, a total of 2865 proteins were identified in the *E. tenella* oocyst wall, which accounted for about 33% (2865/8603) of the total predicted proteins [2]. Two tyrosine-rich proteins, *Et*GAM56 (ETH_00007320) and sporocyst wall protein (*Et*SWP1, ETH_00000115), were detected, but the differences were not significant between SOW vs. Spo. GAM56 is processed into smaller peptides prior to incorporation in the oocyst wall, and these proteolytic products appear to be involved in the formation of intermolecular dityrosine bonds, which is thought to be an essential reaction in the formation of the oocyst wall to contribute to the resilience of oocysts [12,16]. *Et*SWP1 is an intrinsically disordered protein with tyrosine residues concentrated in a specific region of the protein and localized specifically to sporocyst walls [10]. These results indicate that *Et*GAM56 and *Et*SWP1 are present in both oocyst walls and sporocysts; they may be involved in the formation of the oocyst and sporocyst walls of *E. tenella*.

Another type of identified oocyst wall proteins are cysteine-rich proteins, which were originally identified and characterized from *Cryptosporidium parvum* (the so-called COWPs) [19]. Nine COWPs have been identified in *Cryptosporidium* (COWP1–COWP9) [23,24]. COWPs contain different motifs (type I and type II) with cysteine residues in conserved and regularly spaced positions [19]. Both motifs contain six cysteine residues, but type II motifs are shorter than type I motifs and alternated by histidine stretches of variable length. The cysteine residues form disulfide bridges among each other and are responsible for the stabilization and formation of the oocyst wall [19]. In the present study, two OWPs, *Et*OWP (ETH_00012470) and *Et*COWP (ETH_00025735), which showed homology to OWP6 from *Toxoplasma gondii* [25] and *E. nieschulzi* OWP2 [24] and were named “*Et*OWP6 and *Et*OWP2”, respectively, were found to be up-regulated in the oocyst wall. *Et*OWP6 has type II motifs and was previously found in the outer layer of the oocyst wall and the type I wall-forming bodies of macrogametes [20]. *Et*OWP2 has type I motifs. These results suggest that *Et*OWP6 and *Et*OWP2 are important molecules in the oocyst wall and may play important functional roles in the development of *Eimeria* oocyst walls.

Dityrosine is the main enriched amino acid in the oocyst wall, which is indispensable for the development of the oocyst wall. Proteins with oxidoreductase activity are potentially capable of cross-linking dityrosine, and the cross−linking of dityrosine-bonds helps coccidioides to form oocyst walls, which further enhances the stability of oocyst walls [26] and contribute to their resistance to chemical and environmental insult [12]. In the present study, five proteins with predicted oxidoreductase activity were detected in up-regulated DEPs, namely, opine dehydrogenase (ETH_00032295), TPR domain-containing protein (ETH_00027970), dihydrolipoyl dehydrogenase protein (ETH_00041205), oxidoreductase (ETH_00033360), and a hypothetical protein (ETH_00015485). These enzymes with oxidoreductase activity may catalyze the cross-linking of dityrosine during the formation of the oocyst wall, promote the synthesis or decomposition of some key oocyst wall proteins, and thus ensure the integrity and hardness of the oocyst wall structure.

Studies in *T. gondii*, *E. maxima* and *E. tenella* found that lipids in the outer layer of oocyst and sporocyst walls mainly included cholesterol and acid-fast lipids with polyhydroxy fatty acyl chains [9,27,28,29]. Polyketide synthases related to mycobacterial wall lipids have also been detected in abundance in *T. gondii* and *E. tenella* oocysts [27]. Lipid coating could reinforce the robustness of the oocyst wall facing harsh external conditions and contribute to the impermeability of the coccidian oocysts to water-soluble molecules, including disinfectants and detergents [30]. Therefore, fatty acid metabolism-related proteins play an important role in the formation of the oocyst wall and in protecting oocysts from the external environment. In the present study, several enzymes involved in fatty acid degradation, including equisetin synthetase (ETH_00015480), acetyl-CoA carboxylase (ETH_00009260), 3-oxoacyl-(acyl-carrier-protein) synthase III family protein (ETH_00002595), 3-oxoacyl-(acyl-carrier-protein) synthase II (ETH_00005790), fatty acyl-CoA desaturase (ETH_00013640), and a hypothetical protein (ETH_00005790), were found to be up-regulated. Furthermore, several other enzymes, such as alanine dehydrogenase (ETH_00015095), glycan synthetase (ETH_00030295), pyruvate dehydrogenase (ETH_00020990), cytochrome c oxidase subunit (ETH_00011460), and some protein kinases, have been identified in this study. Previous studies found that these enzymes were mainly localized in sporozoites or merozoites, participating in the energy and metabolism of parasites [31,32]. The function of these enzymes in oocyst wall formation needs to be further studied.

Through enriched GO and KEGG pathway enrichment analyses, many nucleic acid metabolic, DNA metabolic/binding, and fatty acid metabolism-related proteins were identified in the up-regulated DEPs. Nucleic acid metabolism can dynamically regulate the quantity and quality of nucleic acids, participating in parasite growth, development, and inheritance [33]. DNA metabolic/binding may have unspecified roles in gametocyte-specific gene regulation [20]. Fatty acid metabolism regulates membrane lipid homeostasis [34,35]. Hence, we speculate that the formation of oocyst walls may involve complex biological processes, resulting from the interaction of multiple proteins and pathways.

Previous studies reported that most proteasome-related proteins are involved in energy metabolism, protein synthesis, and excystation [36]. Analysis of the proteomic composition of *Cryptosporidium* oocyst walls revealed that the oocyst wall proteins were enriched in the proteasome pathway. Many proteasome-related proteins were also detected in a previous RNA-Seq analysis of *E. tenella* [20]. However, in the present, no proteasome pathway enrichment was found in the up-regulated DEPs, but three proteasome-related proteins, subtilisin-like protein (ETH_00005950), ubiquitin carboxyl-terminal hydrolase isozyme L5 (ETH_00002580), and subtilisin 4 (ETH_00006825), were detected. Among the down-regulated DEPs, the proteasome pathway was enriched and 12 related proteins were involved. Proteasome-related proteins play a significant role in the differentiation and invasion stages of coccidian parasites and can be used as virulence factors [37], but their role in oocyst wall formation requires further study.

Other common functional proteins were also found among up-regulated DEPs, such as heat shock protein 90, GRA9, elongation factors, SAG family member, a PAN domain-containing protein (ETH_00027460), and an AN/Apple domain protein (ETH_00012815). These functional proteins are involved in various biological processes, such as the initial attachment of host cells, maintenance of cell homeostasis, invasion, and vacuolar escape [38,39,40,41]. It has also been reported that these proteins may be involved in the development and formation of the oocyst wall. For example, small dense granules cross-react with antibodies to the AN/Apple domains of *T. gondii* microneme protein 4 (*Tg*MIC4) [42], giving rise to the outer veil of early oocysts in *T. gondii* [43]. PAN domain-containing proteins are known to play a role in protein–protein and protein–carbohydrate interactions [44]. The structural conformation of PAN-domain-containing proteins is achieved through disulfide bridges resulting in a pattern of folding that creates recognition and binding sites [45]. In addition, it is possible that the PAN domain-containing proteins in the wall are of structural significance given their large size and predicted disulfide bridges. The role of heat shock proteins, surface antigens, and elongation factors in oocyst wall biology remains to be investigated.

In summary, functional analysis of oocyst wall proteins will help to understand the molecular basis of oocyst wall formation. In this study, a TMT-labeled quantitative proteomics approach led to the identification of hundreds of proteins specifically expressed in *E. tenella* oocyst walls. These data set represents a snapshot of the mechanisms at play in coccidian oocyst wall biosynthesis and also highlights previously undescribed transmission-blocking targets, in particular, four oocyst wall proteins, proteasome-related proteins, and an oxidoreductase. Of the identified DEPs, 96 have not been previously found in oocyst walls. The present study represents a crucial first step to dissect this under-studied but highly important stage in the life cycle of *E. tenella*.

## 4. Materials and Methods

### 4.1. Ethics Statement

The experimental protocol and all associated animal studies were in accordance with the animal care guidelines and approved by the Ethics Committee of the Shanghai Veterinary Research Institute, Chinese Academy of Agricultural Sciences.

### 4.2. Animals and Parasites

Coccidia-free, 1-day-old chickens were kept in a heat-treated coccidia-free house and offered basal feed and cold boiled water. The *E. tenella* Shanghai strain was maintained in our lab and propagated by passage through coccidia-free, 2-week-old chickens as previously described [46]. Unsporulated oocysts were collected from the ceca of 14-day-old chickens infected with 1 × 10^4^ sporulated oocysts at 7 days post-infection. Unsporulated oocysts were suspended with 2.5% potassium dichromate solution and incubated at room temperature until 90% of unsporulated oocysts sporulated. Sporulated oocysts were purified and decontaminated using standard procedures [47,48].

### 4.3. Isolation of Oocyst Walls and Sporocysts

Oocyst walls and sporocysts were isolated from sporulated oocysts as previously described with modifications [47]. Briefly, purified sporulated oocysts were homogenized for 3–5 min by vortexing with an equal volume of glass beads (710–1180 μm, Sigma-Aldrich, St. Louis, MO, USA) until >90% of oocysts were broken. Fragmented oocysts were resuspended in 50% Percoll in phosphate-buffered saline and centrifuged at 11,000× *g* for 2 min. Oocyst walls were harvested at the top of 50% Percoll and the sporocysts were harvested at the pipe bottom. Walls and sporocysts were washed three times in PBS and verified by microscopic examination under a light microscope.

### 4.4. Protein Extraction and Tandem Mass Tag Labeling

Tandem mass tag (TMT) analysis was conducted and total protein was extracted from sporulated oocyst walls (SOW) and sporocysts (Spo) of *E. tenella* with three biological replicates as previously described [49,50,51]. Briefly, the samples were broken by glass beads through vortexing and lysed with DB lysis buffer (8 M Urea, 100 mM TEAB, pH 8.5), followed by 5 min of ultrasonication on ice. The lysate was centrifuged at 12,000× *g* for 15 min at 4 °C and the supernatant was reduced with 10 mM DTT for 1 h at 56 °C, and subsequently alkylated with sufficient iodoacetamide for 1 h at room temperature in the dark to ensure that the protein samples were fully denatured and remained in the reductive state. The protein concentration was determined using the Bradford assay.

Each protein sample was labeled using a TMT reagent (Thermo Fisher Scientific, Shanghai, China) according to the manufacturer’s instructions. All labeling samples were mixed with an equal volume, desalted, and lyophilized. Three technical replicates were performed.

### 4.5. Separation of Fractions and LC-MS/MS Analysis

Mobile phase A (2% acetonitrile, pH adjusted to 10.0 using ammonium hydroxide) and mobile phase B (98% acetonitrile) were used to develop a gradient elution. The lyophilized powder was dissolved in solution A and centrifuged at 12,000× *g* for 10 min at room temperature. The sample was fractionated using a C18 column (Waters BEH C18, 4.6 × 250 mm, 5 μm) on a Rigol L3000 HPLC system; the column oven temperature was set to 45 °C. The eluates were monitored at 214 nm, collected at one tube per minute, and finally combined into 10 fractions. All fractions were dried under vacuum, and then, reconstituted in 0.1% (*v*/*v*) formic acid in water.

LC-MS/MS analysis was performed on an Orbitrap Exploris 480 coupled with FAIMS (Thermo Fisher), with a Nanospray Flex™ (ESI) ion source, a spray voltage of 2.1 kV, and an ion transport capillary temperature of 320 °C. Data-dependent acquisition was adopted for mass spectrometry, the FAIMS compensation voltage was set at −45 and −65 V, respectively, the scan range was from m/z 350 to 1500 with a resolution of 60,000 (at m/z 200), the automatic gain control target value was set at Auto, and the maximum ion injection time was also set at Auto. The scan-round time in MS/MS was set to 1 s, and the precursors in the full scan were selected from high to low abundance and fragmented by high energy collisional dissociation, where the resolution was 30,000 (at m/z 200), the turboTMT + precursor Fit function was turned on, and the automatic gain control target value was 1 × 10^5^. The maximum ion injection time was set at Auto, the normalized collision energy was set as 36%, the intensity threshold was 5.0 × 10^3^, and the dynamic exclusion parameter was 45 s. The raw MS data were stored in a “.raw” file.

### 4.6. The Identification and Quantification of Proteins

The resulting spectra from each run were searched separately against the database accessed on 16 July 2022 (https://toxodb.org/common/downloads/Current_Release/EtenellaHoughton/) by the Proteome Discoverer 2.4 (Thermo) search engine. In order to improve the quality of analysis results, the retrieval results were further filtered using Proteome Discoverer 2.4; peptide spectrum matches (PSMs) with credibility of more than 99% were identified as PSMs. Identified proteins contained at least one unique peptide. The identified PSMs and proteins were retained and analysis was performed with a false discovery rate of no more than 1.0%. The protein quantification results were statistically analyzed by the *t*-test. The proteins with significantly different levels in different groups (*p* ≤ 0.05 and fold change [FC] ≥ 1.5 or FC ≤ 0.67) were defined as differentially expressed proteins (DEPs).

### 4.7. The Functional Analysis of Proteins and DEPs

Gene Ontology (GO) functional analysis was conducted using the interproscan program against the non-redundant protein database (including Pfam, PRINTS, ProDom, SMART, ProSite, PANTHER) [52], and the databases of COG (Clusters of Orthologous Groups) and KEGG (Kyoto Encyclopedia of Genes and Genomes) were used to analyze the protein family and pathway. DPEs were used for Volcanic map analysis, cluster heat map analysis and enrichment analysis of GO and KEGG [53].

### 4.8. Western Blot

Four DEPs, including ETH_00017510 (heat shock protein, down-regulated), ETH_00006930 (Microneme protein Etmic-2, down-regulated), ETH_00011330 (SERPIN1 protein, down-regulated) and ETH_00009260 (acetyl-CoA carboxylase, up-regulated), and one nonsignificant protein (ETH_00007980, 14-3-3 protein) were selected to validate our TMT results. Proteins from SOW and Spo were extracted using IP cell lysis buffer for Western blot (Beyotime, Haimen, China), followed by Western blot analysis. The protein concentration was determined using a NanoDrop ND-2000 spectrophotometer. The proteins were separated by SDS-PAGE and transferred to polyvinylidene fluoride membranes (Millipore, Burlington, MA, USA). Membranes were blocked and incubated with five primary antibodies from rabbit (prepared in our lab) or monoclonal anti-α-tubulin. Subsequently, the membranes were washed and incubated with goat anti-rabbit IgG or goat anti-mouse IgG (LI-COR Biosciences, Lincoln, NE, USA). At last, the membranes were washed and the target bands were detected by ECL (ShareBio, Shanghai, China). Band intensity was quantified by ImageJ software (https://imagej.net/ij/). 

### 4.9. Statistical Analysis

All the experiments were repeated at least three times. All the data are expressed as means ± SD. Statistical analyses were completed using GraphPad. A *p* < 0.05 was considered statistically significant.

## Figures and Tables

**Table 1:** Top 30 oocyst wall fractions proteins in abundance values.

Gene	Description	Average Abundance ^1^	*p* Value	UP.DOWN
ETH_00015095	alanine dehydrogenase	5415	0.00158	up
ETH_00030295	glycan synthetase	3486.767	2.87 × 10^−5^	up
ETH_00000160	acetyltransferase domain-containing protein	3142.4	6.51 × 10^−5^	up
ETH_00015875	Peptidyl-prolyl cis-trans isomerase d-like protein	3116.1	0.000129	up
ETH_00041205	dihydrolipoyl dehydrogenase protein	3070.6	0.000281	up
ETH_00037325	protein disulfide-isomerase	1961.8	0.000482	up
ETH_00040975	histidyl-tRNA synthetase	1749.767	0.000109	up
ETH_00028630	CMGC kinase, CK2 family	1739.9	0.000105	up
ETH_00011615	pseudouridylate synthase 1	1613	0.002113	up
ETH_00020990	pyruvate dehydrogenase	1611.6	0.000588	up
ETH_00036640	alkyl sulfatase	1519.633	0.011512	up
ETH_00005785	PKSN polyketide synthase for alternapyrone biosynthesis protein	1288.9	0.000935	up
ETH_00021530	ribonucleotide-diphosphate reductase, small subunit	714.9333	0.002011	up
ETH_00001040	AGC kinase	694.7667	0.002582	up
ETH_00026125	bifunctional dihydrofolate reductase/thymidylate synthase	668.4667	0.000152	up
ETH_00035460	Lipoyl synthase, chloroplastic	551.5667	0.003634	up
ETH_00023085	SNF2 family helicase	508.3	0.000468	up
ETH_00028615	AGC kinase	465.6333	0.000302	up
ETH_00005380	CMGC kinase, MAPK family	459.8333	0.000614	up
ETH_00011460	cytochrome c oxidase subunit	449.5333	0.001913	up
ETH_00019830	Phosphorylase family protein	389.9333	0.000733	up
ETH_00006545	N2,N2-dimethylguanosine tRNA methyltransferase	357.8333	5.98 × 10^−5^	up
ETH_00033505	D-3-phosphoglycerate dehydrogenase	290.8667	0.000928	up
ETH_00029870	4-nitrophenylphosphatase	143.8333	0.001741	up
ETH_00002930	gamma-glutamylcysteine synthetase	80.03333	0.008645	up
ETH_00035130	Serine/threonine protein kinase	10,300.33	0.000169	up
ETH_00016690	ribonucleoside-diphosphate reductase subunit M2	1412.4	0.00039	up
ETH_00042280	5’-3’ exoribonuclease 2	528.5667	0.014544	up

^1^ Average abundance = sample average value of three repeated biology abundance.

**Table 2:** Up-regulated DEPs proteins involved in the biosynthesis of the oocyst walls.

Gene	Description	Average Abundance ^1^	Function
ETH_00005950	Subtilisin-like protein	4694.6	serine-type endopeptidase activity, proteolysis
ETH_00002580	ubiquitin carboxyl-terminal hydrolase isozyme L5	1556.633333	thiol-dependent ubiquitin-specific protease activity
ETH_00006825	Whole genome shotgun assembly, reference scaffold old set, scaffold scaffold_12 (subtilisin 4)	896.4666667	serine-type endopeptidase activity, proteolysis
ETH_00032295	opine dehydrogenase	6142.566667	oxidoreductase activity
ETH_00027970	TPR domain-containing protein	6142.566667	oxidoreductase activity
ETH_00041205	dihydrolipoyl dehydrogenase protein	3070.6	oxidoreductase activity
ETH_00015485	hypothetical protein	3908.533333	Flavin-dependent oxidoreductase, luciferase family
ETH_00033360	oxidoreductase	1971.266667	oxidoreductase
ETH_00015480	Equisetin synthetase	113,994.1667	Fatty acid metabolism
ETH_00009260	acetyl-CoA carboxylase	14,813.36667	Fatty acid metabolism
ETH_00005790	hypothetical protein	10,591.33333	Fatty acid metabolism
ETH_00002595	3-oxoacyl-(acyl-carrier-protein) synthase III family protein	1044.933333	Fatty acid metabolism
ETH_00005790	3-oxoacyl-[acyl-carrier-protein] synthase II	10,591.33	Fatty acid metabolism
ETH_00013640	fatty acyl-CoA desaturase	731.6666667	Fatty acid metabolism

^1^ Average abundance = sample average value of three repeated biology abundance.

**Table 3:** Other enzymes highly enriched in the oocyst walls fractions.

Gene	Description	Average Abundance ^1^	*p* Value	UP.DOWN
ETH_00015095	alanine dehydrogenase	5415	0.00158	up
ETH_00030295	glycan synthetase	3486.767	2.87 × 10^−5^	up
ETH_00000160	acetyltransferase domain-containing protein	3142.4	6.51 × 10^−5^	up
ETH_00015875	Peptidyl-prolyl cis-trans isomerase d-like protein	3116.1	0.000129	up
ETH_00041205	dihydrolipoyl dehydrogenase protein	3070.6	0.000281	up
ETH_00037325	protein disulfide-isomerase	1961.8	0.000482	up
ETH_00040975	histidyl-tRNA synthetase	1749.767	0.000109	up
ETH_00028630	CMGC kinase, CK2 family	1739.9	0.000105	up
ETH_00011615	pseudouridylate synthase 1	1613	0.002113	up
ETH_00020990	pyruvate dehydrogenase	1611.6	0.000588	up
ETH_00036640	alkyl sulfatase	1519.633	0.011512	up
ETH_00005785	PKSN polyketide synthase for alternapyrone biosynthesis protein	1288.9	0.000935	up
ETH_00021530	ribonucleotide-diphosphate reductase, small subunit	714.9333	0.002011	up
ETH_00001040	AGC kinase	694.7667	0.002582	up
ETH_00026125	bifunctional dihydrofolate reductase / thymidylate synthase	668.4667	0.000152	up
ETH_00035460	Lipoyl synthase, chloroplastic	551.5667	0.003634	up
ETH_00023085	SNF2 family helicase	508.3	0.000468	up
ETH_00028615	AGC kinase	465.6333	0.000302	up
ETH_00005380	CMGC kinase, MAPK family	459.8333	0.000614	up
ETH_00011460	cytochrome c oxidase subunit	449.5333	0.001913	up
ETH_00019830	Phosphorylase family protein	389.9333	0.000733	up
ETH_00006545	N2,N2-dimethylguanosine tRNA methyltransferase	357.8333	5.98 × 10^−5^	up
ETH_00033505	D-3-phosphoglycerate dehydrogenase	290.8667	0.000928	up
ETH_00029870	4-nitrophenylphosphatase	143.8333	0.001741	up
ETH_00002930	gamma-glutamylcysteine synthetase	80.03333	0.008645	up
ETH_00035130	Serine/threonine protein kinase	10300.33	0.000169	up
ETH_00016690	ribonucleoside-diphosphate reductase subunit M2	1412.4	0.00039	up
ETH_00042280	5’-3’ exoribonuclease 2	528.5667	0.014544	up

^1^ Average abundance = sample average value of three repeated biology abundance.

**Table 4:** Common functionally related proteins identified in the oocyst walls fractions.

Gene	Description	Average Abundance ^1^	*p* Value	UP.DOWN
ETH_00040825	heat shock protein 90kDa beta	8598.533	0.004669	up
ETH_00028350	GRA9 protein	4837.967	0.000294	up
ETH_00028100	Eukaryotic translation initiation factor 3 subunit A	2394.167	0.001605	up
ETH_00019335	protein antigen	2165.633	0.000389	up
ETH_00013135	SAG family member	81.63333	0.00222	up
ETH_00014565	elongation factor 1-alpha	54.3	0.042969	up
ETH_00012815	hypothetical protein (AN/Apple domain)	543.6333	0.002164	up
ETH_00027460	PAN domain-containing protein	2479.267	0.001279	up
ETH_00006825	Whole genome shotgun assembly, reference scaffold old set, scaffold scaffold_12 (EGF-like calcium-binding domain)	845.3667	0.001102	up
ETH_00029115	UDP-N-acetyl-D-galactosamine:polypeptide N-acetylgalactosaminyltransferase T2 (lectin domain)	5568.333	0.000111	up

^1^ Average abundance = sample average value of three repeated biology abundance.

**Table 5:** Functionally interesting proteins identified in sporocyst/sporozoite fractions.

Gene	Description	Average Abundance ^1^	*p* Value	UP.DOWN
ETH_00008685	SAG family member	6059.2	9.77 × 10^−5^	down
ETH_00017510	heat shock protein	4203.9	0.000845	down
ETH_00021655	Micronemal protein MIC4	66,229.83	0.000583	down
ETH_00026625	microneme protein 2	25,170.7	0.000718	down
ETH_00024085	Microneme protein 4	25,612.6	0.001731	down
ETH_00021010	microneme protein	14,041.6	0.002008	down
ETH_00006930	Microneme protein Etmic-2	19,872.93	0.008696	down
ETH_00007745	apical membrane antigen-1	5813.8	8.97 × 10^−5^	down
ETH_00017540	microneme protein MIC3	2875.933	0.002346	down
ETH_00000645	thrombospondin type 1 domain-containing protein	361.0667	0.006292	down
ETH_00027625	serine/threonine protein phosphatase	1545.967	0.001134	down
ETH_00024540	calcium-dependent protein kinase	7274.733	0.000642	down
ETH_00011830	Superoxide dismutase	7096.533	0.00051	down
ETH_00028715	CMGC kinase, GSK family TgPK3	4870.3	0.000163	down

^1^ Average abundance = sample average value of three repeated biology abundance.

**Table 6:** Western Blot Verification of the proteomic data.

Gene	Description	SOW.vs. Spo UP.DOWN
ETH_00006930	Microneme protein Etmic-2	down
ETH_00011330	SERPIN1 protein	down
ETH_00007980	14-3-3 protein	NA
ETH_00017510	heat shock protein	down
ETH_00009260	acetyl-CoA carboxylase	up

## Data Availability

Data are contained within the article and Supplementary Materials.

## Supplementary Materials

The following supporting information can be downloaded at: https://www.mdpi.com/article/10.3390/ijms242317051/s1.
